# Various Cellular Components and Its Signaling Cascades Through the Involvement of Signaling Messengers in Keratinocyte Differentiation

**DOI:** 10.3390/antiox14040426

**Published:** 2025-04-01

**Authors:** Hyeong Jae Kim, Dongki Yang, Jeong Hee Hong

**Affiliations:** Department of Physiology, Lee Gil Ya Cancer and Diabetes Institute, College of Medicine, Gachon University, 155 Getbeolro, Yeonsu-gu, Incheon 21999, Republic of Korea; lilili1125@naver.com

**Keywords:** keratinocyte differentiation, skin homeostasis, calcium, oxidative stress

## Abstract

Skin is a highly differentiated tissue, in which various signaling molecules play critical roles in the differentiation and proliferation of keratinocytes. Among these, the second messenger calcium and its gradient across skin layers are pivotal in regulating keratinocyte differentiation. Additionally, a diverse array of cellular signaling molecules has been identified as essential for promoting keratinocyte differentiation, thereby maintaining skin integrity and barrier function. The barrier function of the skin provides essential protection against exogenous stimuli and pathogens while maintaining structural stability. The homeostatic processes of skin differentiation are modulated by these second messengers and various signaling molecules. Thus, this review highlights the components associated with keratinocyte differentiation and their biological and pathophysiological roles, as well as redox-sensitive differentiation factors in the modulation of skin homeostasis. This review aims to enhance our understanding of skin physiology and provide insights that may facilitate the development of novel therapeutic strategies for skin diseases.

## 1. Introduction

Skin is a highly specialized tissue composed of two main layers: the epidermal and dermal layers. The normal epidermis exhibits a distinctive calcium gradient, with low calcium concentrations in the basal layer, increasing gradually towards the granular layer, and diminishing again in the cornified layer [1]. This calcium gradient is crucial for maintaining normal epidermis layer differentiation, as the disruption of the epidermal barrier and consequent loss of the calcium gradient lead to enhanced proliferation and reduced differentiation of keratinocytes [2]. An aberrant calcium gradient has been observed in psoriatic epidermis, which is characterized by excessive proliferation and defective differentiation [3].

**Figure 1:**
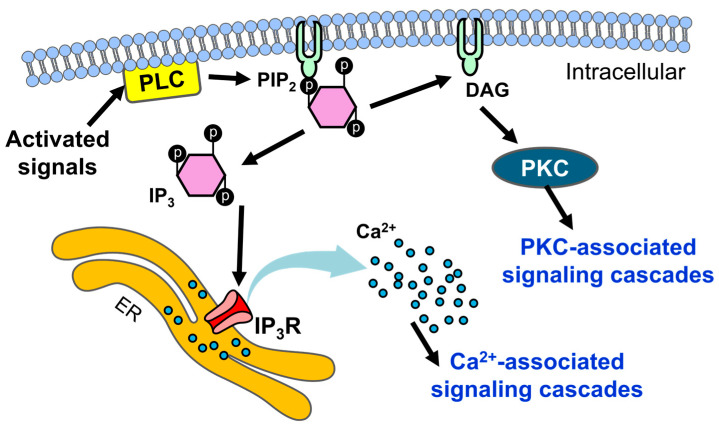
A schematic representation of the signals activated in intracellular calcium signaling and subsequent cellular functions in keratinocytes. Extracellular stimuli trigger the activation of PLC, leading to the hydrolysis of PIP_2_ and the generation of IP_3_ and DAG. IP_3_ binds to IP_3_Rs, resulting in the release of calcium from the ER, while DAG activates PKC. Both calcium and DAG trigger intrinsic cellular signaling pathways that affect various cellular functions [4,5,6,7]. Black arrows represent signaling flow. Light blue arrow represents the release of calcium. PLC: phospholipase C; PIP_2_: phosphatidylinositol bisphosphate; IP_3_: inositol-1,4,5-triphosphate; DAG: diacylglycerol; IP_3_R: inositol-1,4,5-triphosphate receptor; ER: endoplasmic reticulum; PKC: protein kinase C.

Calcium serves as a secondary messenger in cellular signaling and participates in various cellular functions. Various extracellular stimuli such as activated G protein-coupled receptor (GPCR) activate the isozymes of phospholipase C (PLC). The activation of PLC catalyzes the hydrolysis of phosphatidylinositol bisphosphate (PIP_2_), a plasma membrane phospholipid, producing inositol trisphosphate (IP_3_) and diacylglycerol (DAG). IP_3_ binds to inositol-1,4,5-triphosphate receptors (IP_3_Rs) localized in the ER, facilitating the release of calcium from intracellular stores, while DAG activates protein kinase C (PKC) to evoke intracellular signaling cascades. Both calcium and DAG act as second messengers and initiate a range of cellular processes, including the regulation of proliferation and differentiation [4,5,6,7] (Figure 1).

Given the critical role of calcium and other signaling molecules in epidermal homeostasis, this review explores the signaling components associated with keratinocyte differentiation and redox-sensitive differentiation, and discusses their biological and pathophysiological roles in modulating skin homeostasis. This review encompasses evidence sourced from PubMed- and Scopus-indexed literature, aiming to enhance the current understanding of skin physiology and their potential implications for therapeutic strategies in skin disorders.

## 2. Signaling Molecules for Keratinocyte Differentiation

### 2.1. Phospholipase C and Protein Kinase C

Increased calcium levels are crucial for keratinocyte differentiation. Elevated calcium concentrations above 0.1 mM enhance the expression of keratinocyte differentiation markers, such as involucrin (INV) and transglutaminase, through PLC-γ1 signaling pathways in keratinocytes [8]. Enhanced calcium stimulation upregulates PLC-γ1 activity, as well as the expression levels of PIP_2_, phosphatidylinositol 3,4,5-triphosphate (PIP_3_), and IP_3_ via phosphatidylinositol-4-phosphate 5-kinase 1α (PIP5K) 1α activity in human keratinocytes [9]. Additionally, depletion of PIP5K1α in calcium-stimulated human keratinocytes leads to a decrease in calcium release from intracellular stores and downregulates PIP_2_ levels [9]. Increased calcium-induced PIP5K1α activation is mediated by epithelial-cadherin (E-cadherin) and β-catenin in human keratinocytes [9]. Keratinocyte differentiation markers, such as INV, keratin 1, and transglutaminase 1, are upregulated by elevated calcium levels via the involvement of PIP5K1α in human keratinocytes [9].

The activation of PKC during calcium-induced differentiation is essential for the expression of the granular layer differentiation [10,11]. Activation of specific PKC isoforms is implicated in modulating the expression of keratinocyte differentiation genes [10,12,13,14,15]. Calcium stimulation enhances differentiation markers such as keratin (K)1, K10, filaggrin (FLG), and loricrin (LOR), as well as tyrosine phosphorylation of PKC-δ during mouse keratinocyte differentiation [16]. Increased extracellular calcium above 0.1 mM boosts the expression of transforming growth factor (TGF)-α, which in turn promotes phosphorylated (p)-tyrosine of PKC-δ in primary mouse keratinocytes [16]. Activation of the epidermal growth factor receptor (EGFR) induces p-tyrosine of PKC-δ in keratinocytes [17]. The differentiated epidermis relies on p-tyrosine of PKC-δ [16]. In addition to p-PKC-δ, phosphorylated Src homology 2 domain-containing transforming protein, a growth factor receptor adapter protein, is present in differentiated keratinocytes [16].

Moreover, phosphatidylinositol 3-kinase (PI3K) activation requires keratinocyte differentiation [18,19]. PI3K catalyzes the conversion from PIP_2_ to PIP_3_, which subsequently binds to the pleckstrin homology-containing domain at the N-terminal [20] and the src homology 2-containing domain at the C-terminal [21,22] of PLC-γ1 to activate PLC-γ1 [18]. Multiple studies have indicated that PI3K is recruited by E-cadherin, which induces the contact regions of cell–cell to engage with its cell membrane substrate, PIP_2_ [23,24,25,26,27]. During calcium-induced differentiation of murine keratinocytes, enhanced PI3K activity is associated with the E-cadherin–catenin protein complex [27]. An increase in calcium concentration prompts the formation of the E-cadherin–catenin complex at the cellular surface through the recruitment of the PI3K-p85α subunit in human keratinocytes [19]. Within the E-cadherin-associated catenin members, β-catenin has been shown to bind directly to PI3K-p85α [25,28]. In addition to β-catenin, p120-catenin is crucial for the overall stability of the complex [19]. Calcium stimulation-mediated downregulation of E-cadherin expression by p120-catenin knockdown disrupts the stabilization of p120-catenin [19,29,30]. Consequently, E-cadherin and related catenin components such as β-catenin and p120-catenin are critical for the calcium-induced activation of the PLC-γ1/PI3K/Akt serine/threonine kinase (Akt) pathway, which enhances the expression of INV, TGM1, and K1 and leads to keratinocyte differentiation [19]. Thus, verification of the PLC-γ1/PI3K/Akt signaling pathway should be considered as one of the criteria to modulate keratinocyte differentiation.

### 2.2. 1,25-Dihydroxyvitamin D3

1,25-Dihydroxyvitamin D_3_ [1,25(OH)_2_D_3_] is the physiologically active form of vitamin D_3_, synthesized in the epidermis layer, and plays a vital role in calcium homeostasis [31,32]. In addition to its regulatory role in the immune system, 1,25(OH)_2_D_3_ is crucial for modulating changes in function and differentiation in the epidermis layer [33]. While keratinocytes possess receptors for 1,25(OH)_2_D_3_ and naturally produce 1,25(OH)_2_D_3_ [34,35,36,37], the administration of 1,25(OH)_2_D_3_ induces PLC-γ1 activation and upregulates differentiation promoter activity in cultured keratinocytes [33,38]. Furthermore, 1,25(OH)_2_D_3_, together with extracellular calcium, enhance the expression of PLC isoforms such as PLC-β1, -γ1, and -δ1, and increase intracellular calcium and IP_3_, and subsequently induce keratinocyte differentiation [39,40,41]. Accordingly, 1,25(OH)_2_D_3_ is useful for promoting keratinocyte differentiation in experimental procedures and its clinical application should be encouraged in future studies.

### 2.3. Phosphoprotein Phosphatase 1

Phosphoprotein phosphatase 1 (PP1) is a critical member of the serine/threonine phosphatase group within the PP enzyme family [42].It is expressed extensively in mammalian tissues [43,44,45,46,47]. Among the four primary isoforms (PP1, PP2A, PP2B, and PP2C), PP1 and PP2A are the two major enzymes that modulate a wide range of cellular reactions, including glycogen metabolism, muscle contraction, calcium transport, protein synthesis, metabolism, synaptic transmission, RNA splicing, cell cycle progression, and signal transduction [48,49,50]. Generally, PP1 is considered a pivotal regulator of IP_3_R-dependent calcium signaling [51,52].

Recruitment of PP1 to the E-cadherin–catenin–PIP5K1α complex is regulated in a calcium-dependent manner at the cellular surface of human keratinocytes, promoting PIP5K1α activation, which is critical for the activation of PLC-γ1 and subsequent keratinocyte differentiation [53]. Serine phosphorylation of PIP5K1α and its activity are downregulated by inhibiting the PI3K/PLC/PKC/PP1 axis, which leads to reducing keratinocyte differentiation in calcium-stimulated human keratinocytes [53]. PP1 is essential for keratinocyte differentiation through the recruitment of the E-cadherin–catenin–PIP5K1α complex via the PI3K/PLC/PKC/PP1 signaling pathway. Thus, in addition to the PLC-γ1/PI3K/Akt signaling pathway, the PI3K/PLC/PKC/PP1 signaling pathway should be considered as criteria to modulate keratinocyte differentiation.

### 2.4. Calcium-Sensing Receptor and Its Associated Signaling Molecules

**Figure 2:**
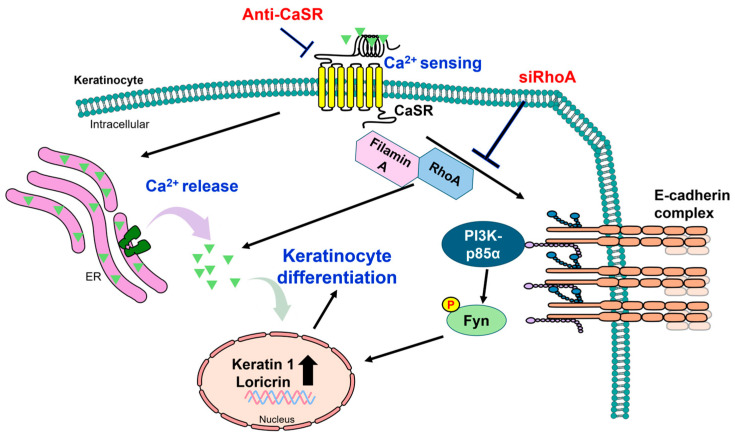
Schematic representation of CaSR-mediated regulation of keratinocyte differentiation. Extracellular calcium binds to the extracellular domain of CaSR, enhancing calcium release from the calcium store ER. In addition, CaSR activation promotes the interaction of E-cadherin complex with RhoA, filamin A, PI3K, and Fyn. Keratinocyte differentiation is upregulated by either an interaction with diverse regulators or an enhanced calcium release [60,61,62,64]. A black bold arrow in the nucleus indicates upregulated protein expression. Light purple and light green arrows represent the release of calcium and calcium-mediated signaling, respectively. CaSR: calcium-sensing receptor; ER: endoplasmic reticulum; Anti-CaSR: CaSR antisense cDNA construct; siRhoA: siRNA-RhoA; RhoA: Ras homolog family member A; PI3K-p85α: phosphoinositide 3-kinase-p85α; Fyn: proto-oncogene tyrosine-protein kinase Fyn; E-cadherin: epithelial-cadherin.

The calcium-sensing receptor (CaSR) as a GPCR responds to variations in extracellular calcium concentrations across various tissues, including the brain, kidneys, bones, and skin [54,55,56,57]. Expression of CaSR has been identified in the epidermis of humans, mice, and rats [58,59]. Elevated intracellular calcium concentrations and subsequent keratinocyte differentiation induced by extracellular calcium stimulation are diminished by the presence of a CaSR antisense cDNA construct (anti-CaSR) in keratinocytes [58]. The proliferative activity of the epidermis is increased in CaSR knockout mice [59]. Additionally, keratinocyte differentiation is reduced in the epidermis of CaSR knockout mice [59]. Binding of calcium to the extracellular domain of CaSR promotes the release of calcium from the intracellular calcium store, such as the endoplasmic reticulum (ER), and subsequently induces keratinocyte differentiation [60,61,62]. CaSR-mediated extracellular calcium stimulation triggers keratinocyte differentiation with the upregulated expression of K1 and LOR in human keratinocytes [63] (Figure 2).

**Figure 3:**
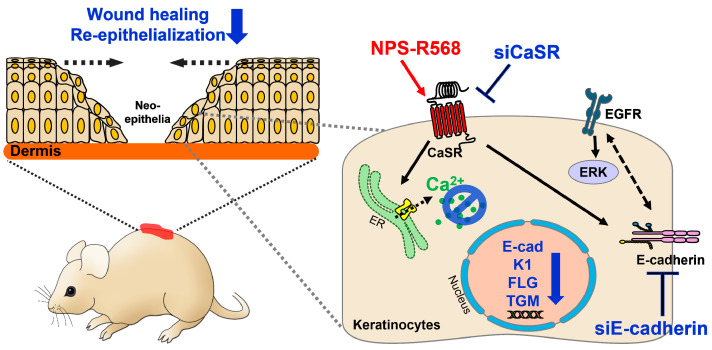
Schematic representation of the inhibition-mediated modulation of keratinocyte differentiation, wound healing, and re-epithelialization in the skin of wounded mice via CaSR inhibition. Keratinocyte differentiation, wound healing, and re-epithelialization are downregulated by decreased intracellular calcium release, interactions between EGFR and E-cadherin, and EGFR/ERK signaling following the treatment with either siCaSR or siE-cadherin. Conversely, the NPS-R568 treatment promotes re-epithelialization [65]. The blue bold arrows denote downregulated protein expression or cellular functions, the red arrow denotes the activating signal, and the dotted arrows indicate the potential mechanism of action. CaSR: calcium-sensing receptor; ER: endoplasmic reticulum; E-cad: epithelial-cadherin; K1: keratin 1; FLG: filaggrin; TGM1: transglutaminase 1; EGFR: epidermal growth factor receptor; ERK: extracellular signal-regulated kinase; siCaSR: siRNA-CaSR; siE-cadherin: siRNA-E-cadherin.

CaSR expression is upregulated in the skin of wounded mice [65]. Depletion of CaSR, intracellular calcium chelation, as well as IP_3_R inhibition, impairs the wound healing process in vitro and in vivo in keratinocyte cultures [65]. The wound-mediated increase in intracellular calcium and the wound healing process are diminished by inhibiting CaSR or E-cadherin with siRNA in keratinocytes [65]. Re-epithelialization, E-cadherin expression, and keratinocyte differentiation are reduced by ablating CaSR in the neo-epithelia of wounded mice skin [65]. Conversely, treatment with the type II calcimimetic NPS-R568, an activator of CaSR, enhances wound re-epithelialization by increasing epidermal calcium signals and promoting membrane localization of E-cadherin [65]. It is well-known that keratinocyte differentiation is stimulated by the EGFR/PI3K/Akt pathway in calcium-activated keratinocytes [27]. In addition, modulation of EGFR signaling plays a role in the regulation of keratinocyte proliferation. Inhibition of CaSR attenuates the interaction between EGFR and E-cadherin, reduces EGFR-mediated extracellular signal-regulated kinase (ERK) activation, and subsequently diminishes keratinocyte proliferation in calcium-activated keratinocytes [65]. Thus, CaSR signaling is implicated in EGFR-mediated cellular adhesion and ERK signaling in keratinocyte differentiation. We have illustrated this summarized information in Figure 3.

Wingless-type MMTV integration site family, member 5A (Wnt5a) is a ligand for members of the frizzled family receptors to induce canonical Wnt signaling. Activation of CaSR, stimulated by extracellular calcium, leads to an intracellular calcium increase, and consequently elevates the expression of Wnt5a, but not those of Wnt3a and Wnt4 [63]. Treatment with Wnt5a dose-dependently downregulates proliferation, while elevated calcium or Wnt5a treatment enhances the expression and activity of Wnt/β-catenin and promotes keratinocyte differentiation in human keratinocytes [63]. Conversely, keratinocyte differentiation is diminished through β-catenin inhibition by siRNA in calcium-stimulated human keratinocytes [63]. Therefore, calcium/CaSR-mediated Wnt5a signaling is essential for keratinocyte differentiation.

Translocations to the cell membrane and subsequent E-cadherin complex formation with various catenin isoforms (α, β, γ, and p120-catenin) and PI3K-p85α are inhibited by anti-CaSR in calcium-stimulated keratinocytes [61]. Activation of CaSR, kinase activity, and E-cadherin complex formation are essential for keratinocyte differentiation. E-cadherin complex formation is inhibited by PP2, an Src family kinase inhibitor, in calcium-stimulated keratinocytes [61]. Furthermore, membrane localization of proto-oncogene tyrosine-protein kinase Fyn (Fyn) is reduced and, subsequently, recruitment of Fyn to the E-cadherin–PI3K complex at the plasma membrane is prevented by calcium-stimulated keratinocytes [61]. Moreover, the small guanosine triphosphatase (GTPase) protein ras homolog family member A (RhoA) plays a role in keratinocyte differentiation. Interactions of E-cadherin with Fyn, tyrosine phosphorylation of Fyn, β, γ, and p120-catenin are reduced by treatment with siRNA-RhoA (siRhoA) in calcium-stimulated keratinocytes [64]. Additionally, inhibition of RhoA by siRhoA treatment decreases basal calcium levels and calcium peaks, thus attenuating keratinocyte differentiation in calcium-stimulated keratinocytes [64] (Figure 2). The interactions of E-cadherin with CaSR, RhoA, and filamin A are augmented in the cell membrane of calcium-stimulated differentiated keratinocytes [64]. In addition to CaSR activation, the Rho activity and interactions of E-cadherin with CaSR, RhoA, and filamin A are essential elements in calcium-stimulated keratinocyte differentiation.

More recently, the CaSR activation is related to the protection from ultraviolet (UV)-induced skin damage. Depletion of CaSR attenuates UV-mediated DNA damage and production of UV-responsive factor cyclobutane pyrimidine dimers in human keratinocytes [66]. Treatment of CaSR antagonist NPS-2143 also attenuates UV-induced skin damages [66]. Although UV-induced differentiation factors have not been identified, potential inhibitory approaches targeting CaSR should be considered in the context of UV-induced skin damages.

### 2.5. Proline-Rich Protein Tyrosine Kinase 2

The proline-rich protein tyrosine kinase 2 (Pyk2), known as a non-receptor tyrosine kinase, is associated with focal adhesion kinases [67]. Serving as an essential integrator in various signaling pathways, Pyk2 is activated by a diverse array of signals including growth factor receptor activations, GPCRs, and environmental stressors. These signals lead to increased intracellular calcium levels and PKC activation, initiating subsequent signaling cascades [68,69,70]. The phosphorylation of Pyk2 enhances its catalytic activity and facilitates the activation of Src family tyrosine kinases [71,72]. Pyk2 orchestrates the regulation of several downstream effectors, including mitogen-activated protein kinase (MAPK) cascades such as ERK1/2, Jun N-terminal kinase (JNK)1/2, p38, p70S6K, Rho family GTPases, Akt, and the nuclear factor kappa-light-chain-enhancer of activated B cells (NF-κB) pathway [68,73,74].

Pyk2 is primarily expressed in the nuclei of keratinocytes [67]. It is established that keratinocyte differentiation is induced by the treatment with 12-O-tetradecanoylphorbol-13-acetate (TPA), a PKC activator, or elevated calcium concentrations [75,76,77,78,79]. The expression of p-Pyk2 is influenced by the involvement of PKC, intracellular calcium, and Src family kinases in TPA or calcium-stimulated keratinocytes [67]. Keratinocyte differentiation is facilitated by the activation of INV promoter activity and PKC in Pyk2-overexpressed or TPA-stimulated keratinocytes [67]. Although a multifaceted role of Pyk2 is present in signaling cascades, phosphorylation of Pyk2 is considered a contributing factor and its specific associated factors should be identified from proteomics research in keratinocyte differentiation.

### 2.6. Activator Protein-1

The activator protein-1 (AP-1) transcription factors consist of homo- or heterodimers composed of members from the FBJ murine osteosarcoma viral oncogene homolog (Fos-B, c-Fos, Fra-1, and Fra-2) and Jun proto-oncogene (JunB, JunD, and c-Jun) protein families [80]. AP-1 plays critical roles in various cellular processes including differentiation, proliferation, apoptosis, and oncogenesis [81,82]. Furthermore, AP-1 is known to regulate skin homeostasis-associated contributors, such as K1, K5, K14, K17, INV, LOR, and proFLG [83,84,85,86,87,88,89]. Among the AP-1 regulators, Fos-related antigen 1 (Fra-1) and JunD are necessary for INV expression in human keratinocytes [90,91,92]. The expressions of Fra-1 and JunD are elevated in TPA and Pyk2-co-stimulated keratinocytes [67]. Additionally, the expressions of Fos proteins, apart from Fos-B, and Jun proteins are shown to occur in a differentiation stage-dependent manner in cultured keratinocytes and in skin equivalents, which are reconstructed human skin cells [93]. Moreover, c-Jun is identified as a positive regulator and JunB as a negative regulator of proliferation in keratinocytes [80,94,95]. Increased activity of JunB and reduced activity of c-Jun are prompted by PKC activation in TPA-stimulated differentiated keratinocytes [96]. Moreover, adipose tissue-associated peptide hormone leptin induces oxidative stress and enhances AP-1 activity in keratinocytes, however, its activity is not sufficient to enhance keratinocyte differentiation [97]. Thus, the multifunctional role of AP-1 in skin homeostasis should be delineated more precisely in the forthcoming years.

A dominant negative form of c-Jun, TAM67, is involved in the modulation of mouse skin phenotypes [98,99,100]. TAM67 induces various epidermal phenotypes including delayed differentiation, increased cell proliferation, extensive parakeratosis, hyperkeratosis, and nuclear LOR accumulation [101]. Additionally, another study has addressed the decreased thickness of the cornified envelope and aberrant formation of keratin filaments, desmosomes, and lamellar body morphology [102]. The composition of the cornified envelope in the epidermal layer of TAM67-transgenic mice demonstrates attenuated levels of late envelope precursor proteins and cutaneous keratins, and hair-related proteins, while exhibiting elevated levels of proline-rich proteins and keratins [102]. Erythema, blood flow, and epidermal thickness are increased in TAM67-transgenic mice [103]. Additionally, T helper type 1 cell (Th)-1- and Th-2-associated chemokine levels are elevated in the serum and epidermal layers of TAM67-transgenic mice [103]. The differentiation markers LOR and FLG are decreased, while expressions of K6, S100A8, and S100A9 are elevated in isolated epidermal tissue from TAM67-transgenic mice [103]. Although the levels of C-X-C motif chemokine receptor 3 (CXCR3) ligands (CXCL9, 10, and 11), S100A8, and S100A9 are elevated in TAM67-transgenic mice [102,103], the depletion of the CXCR3 receptor or S100A8 in TAM67-transgenic mice did not influence the flaked and scaly epidermis phenotype, keratinocyte differentiation, and proliferation [103]. Future studies should investigate TAM67-associated signaling to elucidate its precise role in skin differentiation.

### 2.7. Thrombomodulin

Thrombomodulin (TM), a well-characterized anti-coagulant glycoprotein of the cellular membrane, is expressed in various cell types including epidermal keratinocytes, endothelial cells, leucocytes, the mesothelium, and astrocytes [104,105]. Soluble TM, secreted from cultured keratinocytes, contributes to wound healing by modulating cell proliferation and migration [106]. During the early phase of cutaneous wound healing, enhanced TM expression is observed in the hyperproliferative epithelium of humans and mice [107].

Expression of adhesion molecules and keratinocyte differentiation is enhanced in calcium-stimulated primary keratinocytes from control mice [108]. Conversely, the calcium-induced keratinocyte differentiation is attenuated by downregulated p-ERK expression in TM-depleted keratinocytes [108]. Calcium-stimulated cell migration is reduced in both in vitro and in vivo cultures from TM-depleted mouse models [108]. After recombinant TM-intradermal injection, wound healing is accelerated in TM-depleted mice [108]. These results indicate that TM is essential for keratinocyte differentiation and the recovery of skin wounds. Thus, the clinical implication of TM in differentiation-defective skin diseases should be studied in coming years.

### 2.8. CD9

Cluster of differentiation 9 (CD9), a protein with four transmembrane domains, has been reported to be associated with keratinocyte motility and growth in vitro [109,110]. The expression of CD9 is downregulated at the wound margin and upregulated in the re-epithelialized epidermis of mouse skin [111]. Furthermore, CD9 expression is enhanced in the calcium-stimulated human keratinocytes and primary mouse keratinocytes, as well as differentiated keratinocytes of wounded mouse skin [112].

E-cadherin-mediated cell–cell contacts are associated with the modulation of keratinocyte migration and differentiation [113,114]. E-cadherin-null mice display reduced adherent junctions and impaired differentiation of the epidermis [115,116]. Expression of E-cadherin complexes (e.g., E-cadherin, β-catenin, and p120-catenin) is suppressed by CD9 inhibition in the plasma membrane of calcium-stimulated differentiated mouse keratinocytes [112]. The recruitment of PI3K to the E-cadherin–catenin complex at the cellular membrane and the subsequent phosphorylation of Akt are essential processes for the differentiation of calcium-stimulated keratinocytes [18,19]. The expression of phosphorylated Akt (p-Akt) is upregulated in CD9 overexpression and calcium-stimulated differentiated mouse keratinocytes [112]. Dysfunction of E-cadherin reduces the CD9-mediated expression of p-Akt and disrupts keratinocyte differentiation [112].

**Figure 4:**
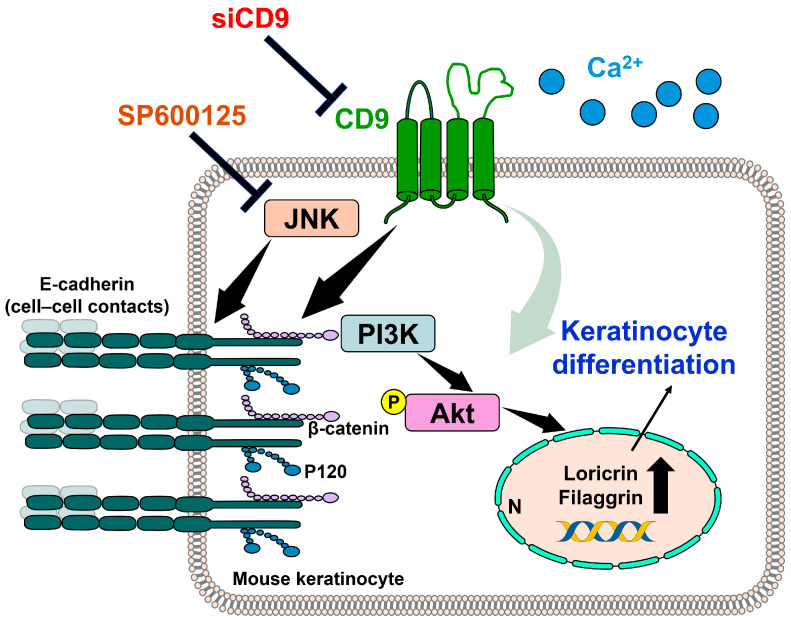
Schematic representation of keratinocyte differentiation mediated by either CD9 overexpression or calcium stimulation in mouse keratinocytes. Differentiation of keratinocytes is enhanced via the PI3K/Akt signaling pathway induced by CD9 overexpression or calcium stimulation [18,19,112]. A black bold arrow in the nucleus indicates upregulated protein expression. Black and light green arrows represent activating signaling. CD9: cluster of differentiation 9; E-cadherin: epithelial-cadherin; JNK: Jun N-terminal kinase SP600125: JNK inhibitor; PI3K: phosphoinositide 3-kinase; Akt: Akt serine/threonine kinase; N: nucleus; siCD9: siRNA-CD9.

Inhibition of the JNK pathway enhances epidermal keratinocyte differentiation [117]. In CD9-depleted mouse keratinocytes, diminished E-cadherin complex expressions are restored through JNK inhibition, which correlates with improved keratinocyte differentiation [112]. Decreased E-cadherin expression, reduced keratinocyte differentiation, and thinning of the epidermal layer result from CD9 depletion in the organotypic model of human keratinocytes and epidermis model of wounded mice [112]. Thus, the CD9/E-cadherin/p-Akt pathway plays a crucial role in regulating keratinocyte differentiation and adhesion. This information is summarized in Figure 4.

### 2.9. microRNA-203

The miRNAs are implicated in skin development in conditional knockout mice of *Dicer*, an enzyme essential for miRNA biogenesis [118]. Notably, microRNA (miR)-203 expression is exclusively detected in human keratinocytes [119]. Intriguingly, miR-203 expression is not consistently observed in normal human epidermis but exhibits a gradient, with increased expression of miR-203 in the more differentiated suprabasal layers and decreased expression in the basal cell layer [119]. Consistent with human data, miR-203 expression is upregulated in the suprabasal layers of normal mouse epidermis [120]. Additionally, decreased epidermal thickness and proliferation are induced in miR-203-overexpressed mice [120]. The expression of miR-203 is upregulated in differentiated keratinocytes stimulated by calcium, TPA, or 1,25(OH)_2_D_3_ [96]. Additionally, expressions of miR-203 and INV are enhanced in keratinocytes at high cell density or through PKC activation in TPA-stimulated keratinocytes [96]. These results suggest that differentiation is mediated via the PKC/miR-203 pathway, characterized by increased JunB activity and reduced c-Jun activity in calcium- or TPA-stimulated keratinocytes [96]. Considering the pharmacological potential of miR-203, its therapeutic applications warrant further investigation in the forthcoming years. Collectively, considering the differentiated role of CaSR, the development of identified relating factors should be encouraged in keratinocyte differentiation.

### 2.10. TGF-β-Inducible Gene-h3

The TGF-β-inducible gene-h3 (β ig-h3) consists of 671 amino acids, and its expression in the extracellular matrix has been detected, induced by TGF-β in various cell types including melanoma, keratinocytes, mammary epithelial cells, and pulmonary adenocarcinoma cells [121,122]. Furthermore, β ig-h3 functions as a cell adhesion protein in fibroblasts [123] and acts as a linking protein connecting multiple matrix proteins [124,125]. The β ig-h3 contains multiple cell adhesion motifs interacting with various integrin complexes such as α3β1 [126,127], α1β1 [128], and αvβ5 [123]. For the skin, β ig-h3 is highly expressed in the papillary dermis and epidermal granular layers [129].

The expression of β ig-h3 and subsequent keratinocyte differentiation are upregulated in TGF-β-stimulated oral and epidermal keratinocytes [130]. Enhanced keratinocyte differentiation and reduced cell proliferation are facilitated by increased promoter activity of INV and transglutaminase, and the involvement of PI3K/Akt signaling in β ig-h3-overexpressed oral keratinocytes, without alterations in intracellular calcium levels [130]. Keratinocyte adhesion is mediated through the involvement of integrin α3β1 in β ig-h3-stimulated oral keratinocytes [130]. Although the promising role of β ig-h3 in oral keratinocyte differentiation is recognized, future studies should further explore its role in normal skin tissue and interactions with other adhesion molecules.

### 2.11. Sphingosine-1-phosphate and Lysophosphatidic Acid

Sphingosine-1-phosphate (S1P) and lysophosphatidic acid (LPA), as lysophospholipids, serve as crucial autocrine and paracrine signaling molecules involved in the regulation of biological processes including survival and growth, differentiation, adhesion, cell motility, and the elevation of intracellular calcium levels in various cell types [131,132,133,134,135,136]. Stimulation with S1P or LPA increases expressions of K1, K10, and INV in human keratinocytes [137]. LPA stimulation induces intracellular calcium spikes via activation of the LPA2 receptor in human keratinocytes [137]. Additionally, S1P stimulation leads to increased intracellular calcium from intracellular stores and simultaneous extracellular calcium influx via the S1P3 receptor in human keratinocytes [137]. Both S1P and LPA trigger PIP_2_ hydrolysis and IP_3_ generation in keratinocytes [137]. Stimulation of the S1P1 receptor by SEW2871, a specific agonist of S1P1, enhances migration without intracellular calcium mobilization in keratinocytes [137], suggesting a therapeutic effect of SEW2871 on the wound healing process.

Intracellular concentrations of S1P are tightly regulated in a spatiotemporal manner through degradation by S1P lyase (SGPL) and specific S1P phosphohydrolases, as well as synthesis via sphingosine kinases (SphKs) [138]. The isoforms SphK-1 and SphK-2 play a role in regulating the relative concentrations of sphingosine, S1P, and ceramide within sphingolipid metabolism [138,139,140]. K6PC-5, characterized as a lipophilic molecule, comprises two short alkyl chains, two hydroxyl groups, a ketone functional group, and an amide bond [141]. SphK activity is increased following the treatment with K6PC-5 in both mouse blood and F9-12 mouse embryonic carcinoma cells [141]. The treatment with K6PC-5 induces an intracellular calcium peak through activation of SphK1 in HaCaT keratinocytes [141]. K6PC-5/SphK1-mediated intracellular calcium peaks result from calcium influx from both intracellular stores and extracellular media, independent of PLC/IP_3_ signaling in HaCaT keratinocytes [141]. Stimulation by K6PC-5 enhances the expression of differentiation markers such as FLG, INV, and K5, through activation of SphK1-S1P signaling in HaCaT keratinocytes and mouse epidermis [141]. Moreover, the K6PC-5 treatment mitigates epidermis hyperplasia through the suppression of keratinocyte proliferation in a mouse model of epidermis hyperplasia [141].

SGPL, a membrane-bound enzyme, irreversibly degrades S1P into phosphoethanolamine and hexadecanal, leading to a reduction in intracellular S1P levels. The S1P protein level is enhanced by upregulated SphK1 in calcium-stimulated keratinocytes [142]. Additionally, keratinocyte differentiation is increased in S1P-stimulated keratinocytes [142]. The S1P protein level is also elevated through the inhibition of SGPL in keratinocytes treated with an SGPL-specific inhibitor or siRNA-SGPL1 [142]. Beyond the in vitro model, psoriasis symptoms and epidermal thickness are alleviated through injection with an SGPL-specific inhibitor in an imiquimod (IMQ)-induced psoriasis mouse model [142]. More recently, keratinocyte differentiation has been upregulated through clustered regularly interspaced short palindromic repeats-associated protein 9 (CRISPR-Cas9)-mediated SGPL1 knockout in keratinocytes and 3D organotypic models [143]. Moreover, thickened stratum corneum and abnormal expression of E-cadherin have been induced through CRISPR-Cas9-based SGPL1 knockout in 3D organotypic models [143]. Collectively, these findings indicate that S1P- and LPA-mediated lysophospholipid signaling play a pivotal role in keratinocyte differentiation.

### 2.12. Serine Protease Inhibitors B7

Abnormal modulation and execution of protease-mediated processes are vital in inducing various human skin pathologies, including Tylosis, Ichthyosis Hypotrichosis syndrome, and Nagashima-type palmoplantar keratosis [144]. Furthermore, these processes influence a broad spectrum of cellular physiological activities such as keratinocyte differentiation, proliferation, desquamation, cornification, and immune system regulation [144]. Serine protease inhibitors (Serpins) are proteins characterized by a conserved tertiary structure associated with diverse cellular functions, encompassing fibrinolysis, cell growth, and inflammation [145]. Serpin B7 is present in the mouse epidermis and exhibits elevated expression in the lesional psoriatic skin of patients [146]. Expression of serpin B7 is upregulated and positively correlated with interleukin-17 in the epidermis of psoriatic lesions and IMQ-stimulated psoriatic-like mouse model [147]. Depletion of serpin B7 results in exacerbated symptoms of psoriasis, increased epidermal thickness, inflammatory infiltration, enhanced chemokine expression, and reduced keratinocyte differentiation in IMQ-stimulated psoriatic-like mouse models [147]. Upregulated expression of chemokines, which influence the pathogenesis and progression of psoriasis, and downregulated keratinocyte differentiation are induced by serpin B7 depletion, which inhibits the intracellular calcium concentration in calcium-stimulated human keratinocytes [147]. These results suggest that serpin B7 depletion impairs keratinocyte differentiation via reduced calcium levels. Expression of serpin B7 is essential for keratinocyte differentiation and the biogenesis of inflammatory chemokines. Currently, the molecular role of serpin B7 in skin biogenesis is poorly understood; thus, the precise effects of serpin B7 on skin diseases should be verified in future studies.

### 2.13. Aquaporin 3

Aquaporins (AQPs), a family of small transmembrane channels, facilitate the transport of water and glycerol [148,149]. AQP3-depleted mice exhibit reduced glycerol and water transport capacities in the epidermal layer, resulting in compromised skin elasticity and delayed barrier recovery [150,151]. Glycerol, a critical component of energy metabolism, plays vital roles in various physiological processes including lipid synthesis, gluconeogenesis, osmoregulation, glucose homeostasis, and apoptosis [152]. AQP3 co-localizes with phospholipase D (PLD)2, which uses glycerol as a substrate in the transphosphatidylation reaction within caveolin-rich membrane microdomains in keratinocytes [153]. Moreover, AQP3 expression and glycerol uptake are downregulated in differentiated keratinocytes, suggesting that the AQP3/PLD2 module is involved in the phosphatidylglycerol (PG) production [153].

PG production from phosphatidylcholine is increased by upregulated PLD activity in calcium-stimulated differentiated keratinocytes [154]. Moreover, PG production is driven by PLD2 activity and PG treatment enhances PKC βII activation in the plasma membrane of primary mouse keratinocytes, and subsequently promotes keratinocyte differentiation [155]. The AQP3/PLD2/PG production axis provides a modulatory module on keratinocyte differentiation through PKC βII activation. Currently, the dominant role of AQP3 including glycerol uptake in keratinocyte differentiation should be identified in the future.

### 2.14. Ephrin-A

Ephrin (Eph) receptors comprise 16 isoforms of Eph receptors (EphA1~A10 and EphB1~B6), which are receptor tyrosine kinases facilitating intercellular communication by interacting with adjacent ephrin-A (EphA1~EphA6) and -B (EphB1~EphB3) ligands [156,157]. The activation and expression of EphA2 are modulated by E-cadherin [158,159,160]. Furthermore, EphA2 activation, triggered by recombinant EphA1 peptide, diminishes the expression levels of ERK1/2 and MAPK, thereby inhibiting proliferation in primary mouse keratinocytes [161]. Additionally, keratinocyte proliferation and colony size are inhibited by stimulating EphA2 activation through recombinant EphA1-Fc peptide in calcium-stimulated keratinocytes [162]. Keratinocyte stratification is associated with desmosomal components, such as desmoglein 1, 3, and desmocollin 1 [163]. Keratinocyte stratification and differentiation are enhanced through the activation of EphA2 mediated by recombinant EphA1-Fc peptide in calcium-stimulated keratinocytes [162]. Additionally, desmoglein 1 promotes keratinocyte differentiation [164]. Inhibition of desmoglein 1 by microRNA-desmoglein 1 diminishes the differentiation of keratinocytes treated with recombinant EphA1-Fc peptide in the presence of calcium [162]. Consequently, the activation of EphA2 represents a favorable approach for enhancing keratinocyte differentiation, and the identification of its associated factors could be a challenging issue.

### 2.15. Insulin-like Growth Factor-Binding Protein 7

Insulin-like growth factor-binding protein 7 (IGFBP7) associates with members of the TGF-β superfamily of growth factors and keratinocyte differentiation [165]. In psoriatic skin, the expression of IGFBP7 is downregulated [165]. Keratinocyte differentiation is attenuated by blocked IGFBP7 expression in calcium-stimulated keratinocytes [165].

Furthermore, IGFBP7 regulates the activity of insulin-like growth factors (IGFs) and insulin [165]. The signaling pathways of insulin and IGF-1 have been demonstrated to promote the proliferation of keratinocytes [166,167] and are involved in skin differentiation [167,168,169]. IGFBP7 is linked with reduced levels of p-ERK1/2 [170] and p-insulin receptor substrate 1 (p-IRS1) [171], which are key components of the insulin signaling pathway. Similarly, the expression levels of p-IRS-1 and p-ERK1/2 are increased in IGFBP7-depleted keratinocytes [165]. The insulin and IGF signaling pathways are implicated in keratinocyte differentiation through the modulation of IGFBP7 levels.

## 3. Redox-Sensitive Differentiation Component

Nuclear factor erythroid 2-related factor 2 (Nrf2) is known as a protection factor for oxidative stress and highly expressed in skin cells such as keratinocytes, melanocytes, and other types of cells [172]. Keratinocyte differentiation is elicited through Nrf2 involvement in calcium-stimulated keratinocytes [173]. Recently, the role of Nrf2 is highlighted in the psoriasis model. The expression and translocation of Nrf2 are promoted through binding to promoter regions of the Nrf2 target gene by treatment with tussilagone (TGN), an anti-inflammatory agent and natural compound isolated from the buds of *Tussilago farfara* in human keratinocytes [174]. The expressions of NF-κB and p-signal transducer and activator of transcription 3 (STAT3) are attenuated through the Nrf2/heme oxygenase-1 pathway by treatment with TGN in TNF-α- or interleukin-6-stimulated human keratinocytes [174]. In addition to in vitro, psoriasis symptoms, keratinocyte proliferation, and expressions of NF-κB and p-STAT3 are reduced by treatment with TGN in the IMQ-induced psoriasis mouse model [174].

Attenuated ROS concentration and enhanced expression and nuclear translocation of Nrf2 occur in anti-psoriatic drug monomethylfumarate (MMF)-treated mouse keratinocytes [175]. Moreover, the Nrf2 is involved in the protection of skin fibrosis [176]. The keratinocyte differentiation is induced through involvement of the Nrf2/AQP3 axis in MMF-treated mouse keratinocytes [175,177]. Whereas prolonged Nrf2 activation induces follicular hyperplasia and keratinized cysts in patients with dioxin-induced skin hamartomas [178]. Thus, Nrf2 could be a negative regulator of oxidative stress, however, Nrf2 is linked to hyperproliferative signals. The relationship between Nrf2 and other differentiation components or the precise role of Nrf2 in skin homeostasis should be clarified in the coming years.

## 4. Concluding Remarks

The regulation of the skin barrier is crucial in protecting against infection and maintaining structural integrity and homeostasis. Keratinocyte differentiation necessitates complex and multifaceted networks comprising various cellular components, such as receptors, kinases, multiple signaling proteins, membrane lipids, vitamin D3, miRNAs, and signaling messengers including intracellular calcium and DAG. Although increased intracellular calcium levels are critical for keratinocyte differentiation, calcium-dependent signaling molecules also play essential roles in this process. We summarized diverse functions of signaling molecules and related physiological states or diseases for keratinocyte differentiation in Table 1. A layer-based differentiation of skin facilitates the finely-tuned modulation of signaling components. In addition, the mechanism of oxidative stress-associated components should be verified to recover skin damage. Therefore, comprehending the intricate interactions among these signaling pathways and their components provides invaluable insights into the pathophysiology of skin diseases and impaired wound healing. Although multifaceted networks are involved, the precise modulation of various signaling components and tight regulation of the proliferation process remain challenging. Identifying components that finely control the differentiation, proliferation, and modulation of oxidative stress are crucial for developing targeted therapeutic strategies to treat skin diseases and enhance wound healing.

The activation of calcium, CaSR, and the PLC/PI3K/PKC/Akt signaling pathways would be effectively modulated by agents for the treatment of chronic wounds and differentiation-defective skin conditions, such as psoriasis or atopic dermatitis. Moreover, oxidative stress is reduced and differentiation is promoted by the antioxidant regulator Nrf2. Furthermore, compounds such as TGN or MMF are recognized as potential therapeutic agents for inflammatory skin disorders. Additional regulators such as miR-203, serpin B7, AQP3, and S1P are involved in keratinocyte differentiation, hydration, and immune modulation, suggesting therapeutic roles in skin barrier restoration, aging skin repair, and wound regeneration. In conclusion, the molecular pathways presented in this review provide a strong scientific foundation for the development of clinical strategies targeting wound healing and skin disease treatment, and may be broadly applicable to future therapeutic innovations in dermatology and regenerative medicine.

## Figures and Tables

**Table 1:** Diverse functions of signaling molecules and related physiological states or diseases for keratinocyte differentiation.

Signaling Molecules	Functions of Signaling Molecules	Related Physiological States or Diseases for Keratinocyte Differentiation	Refs.
Ca^2+^/PIP5K1α/PLC-γ1 or Ca^2+^/PKC-δ	Induction of keratinocyte differentiation	Differentiation of granular layer	[8,9,10,11]
Ca^2+^/PI3K/Akt	Conversion from PIP_2_ to PIP_3_, PLC-γ1 activation, involvement in keratinocyte differentiation, and interaction with the E-cadherin complex	Induction of contact regions of cell–cell	[18,19,20,23,24,25,26,27]
1,25(OH)_2_D_3_/PLC-γ1	Induction of activation and expression of PLC isoform, increase in intracellular Ca^2+^ and IP_3_, and promotion of keratinocyte differentiation	Maintenance of Ca^2+^ homeostasis	[31,32,39,40,41]
PP1	Keratinocyte differentiation through the interaction with the E-cadherin–catenin–PIP5K1α complex via the PI3K/PLC/PKC/PP1 signaling pathway	Cell cycle progression and Ca^2+^ transport	[50,53]
Ca^2+^/CaSR	Ca^2+^ release from intracellular Ca^2+^ store and induction of keratinocyte differentiation	Enhanced epidermis proliferation by CaSR knockout	[59,60,61,62,63]
CaSR/E-cadherin/EGFR/ERK	Induction of keratinocyte differentiation	Wound healing and re-epithelialization	[27,65]
Pyk2	Activation of Src family tyrosine kinases, MAPK, p70S6K, Rho GTPases, Akt, and NF-κB, and keratinocyte differentiation by Pyk2 overexpression	INV promoter activation	[67,68,71,72,73,74]
AP-1	Involvement of differentiation, proliferation, apoptosis, and oncogenesis	Regulation of skin homeostasis	[81,82,83]
TAM67	Induction of delayed differentiation and increased proliferation	Extensive parakeratosis, hyperkeratosis, aberrant formation of keratin filaments, erythema, Th-1- and -2-associated inflammation	[101,103]
TM/p-ERK	Contribution to wound healing and enhanced keratinocyte differentiation	Attenuated cell migration and keratinocyte differentiation by depletion of TM	[106,108]
CD9/E-cadherin/PI3K/Akt	Upregulation of cell adhesion and keratinocyte differentiation	Association with keratinocyte motility and growth	[18,19,109,110,112]
PKC/miR-203	Mediation of keratinocyte differentiation	Decrease in epidermal thickness and proliferation	[96,120]
β ig-h3	Enhanced keratinocyte differentiation	Reduced proliferation and mediation of keratinocyte adhesion	[130]
S1P/S1P3 receptor or LPA/LPA2 receptor	Increase in keratinocyte differentiation through intracellular Ca^2+^ spikes and conversion to IP_3_	Survival and growth, differentiation, adhesion, cell motility, and the elevation of intracellular Ca^2+^ levels	[131,132,133,134,135,136,137]
K6PC-5 or Ca^2+^/SphK/S1P	Induction of keratinocyte differentiation through intracellular Ca^2+^ peaks and suppression of keratinocyte proliferation	Attenuated epidermis hyperplasia	[141,142]
SGPL/S1P	Induction of keratinocyte differentiation by inhibition of SGPL	Alleviation of psoriasis symptoms and epidermal thickness through the SGPL inhibitor	[142,143]
Serpin B7	Increased epidermal thickness, inflammatory infiltration, enhanced chemokine expression, and reduced keratinocyte differentiation by serpin B7 depletion	Exacerbated symptoms of psoriasis	[147]
AQP3/glycerol or AQP3/PLD2/PG/PKC βII	Reduced glycerol and water transport capacities by AQP3 depletion and promotion of keratinocyte differentiation	Compromised skin elasticity delayed barrier recovery	[150,151,155]
Eph A2/desmosomal or Eph A2/ERK/MAPK	Inhibition of keratinocyte proliferation and enhanced keratinocyte stratification and differentiation	Diminished keratinocyte differentiation by desmoglein 1 inhibition	[161,162,163,164]
IGFBP7/p-ERK1/2/p-IRS1 or IGFBP7/IGF/insulin	Attenuated keratinocyte differentiation by blocked IGFBP7 expression	Psoriasis	[165,166,167,168,169,170,171]
Ca^2+^/Nrf2, TGN/Nrf2/HO-1 or MMF/Nrf2/AQP3	Induction of keratinocyte differentiation, alleviation of inflammation, and attenuated ROS level	Psoriasis and skin fibrosis	[173,174,175,176,177]

Abbreviations: PIP5K1α: phosphatidylinositol-4-phosphate 5-kinase 1α; PLC-γ1: phospholipase C- γ1; PKC-δ: protein kinase C-δ; PI3K: phosphatidylinositol 3-kinase; Akt: Akt serine/threonine kinase; PIP_2_: phosphatidylinositol bisphosphate; PIP_3_: phosphatidylinositol 3,4,5-triphosphate; 1,25(OH)_2_D_3_: 1,25-Dihydroxyvitamin D_3_; IP_3_: inositol 1,4,5-trisphosphate; PP1: phosphoprotein phosphatase 1; CaSR: calcium-sensing receptor; E-cadherin: epithelial-cadherin; EGFR: epidermal growth factor receptor; ERK: extracellular signal-regulated kinase; Pyk2: proline-rich protein tyrosine kinase 2; MAPK: mitogen-activated protein kinase; INV: involucrin; AP-1: activator protein 1; TM: thrombomodulin; miR: micro-RNA; β ig-h3: TGF-β-inducible gene-h3; S1P: sphingosine-1-phosphate; LPA: lysophosphatidic acid; SphK: sphingosine kinase; SGPL: S1P lyase; Serpin B7: serine protease inhibitors B7; AQP3: aquaporin 3; PLD2: phospholipase D2; PG: phosphatidylglycerol; PKC βII: protein kinase βII; Eph A2: ephrin A2; IGFBP7: insulin-like growth factor-binding protein 7; p-IRS1: phosphorylate-insulin receptor substrate 1; IGF: insulin-like growth factor; Nrf2: nuclear factor erythroid 2-related factor 2; HO-1: heme oxygenase-1; TGN: tussilagone; MMF: monomethylfumarate; ROS: reactive oxygen species.
